# Perspectives on the Children’s Health Collection 2015

**DOI:** 10.1289/ehp.1511049

**Published:** 2016-01-01

**Authors:** Sally Perreault Darney, Martha M. Dimes

**Affiliations:** 1 Editor-in-Chief and; 2 Children’s Health Editor, *Environmental Health Perspectives*, National Institute of Environmental Health Sciences, National Institutes of Health, U.S. Department of Health and Human Services, Research Triangle Park, North Carolina, USA

*EHP*’s sixth annual Children’s Health Collection, now online at http://ehp.niehs.nih.gov/special-collections, compiles a year’s worth of research, commentary, and news published from October 2014 through September 2015. From the negative health effects of chemical, physical, and social hazards to the benefits of living in a healthy environment—both natural and built—the Collection tells the story of where we are today and points to the important work that lies ahead. It offers something for everyone concerned about children’s environmental health—researchers, regulators, advocates, health care providers, policy makers, educators, community developers, and parents—and we encourage you to download and share it.

Reflecting upon the latest Collection, we are struck by how quickly the field of children’s environmental health is expanding and evolving, leading to a far more holistic understanding of how diverse environmental factors contribute to a child’s growth and development, from before birth through childhood and into adulthood. We are coming to appreciate the value of research designed to integrate children’s environmental exposures and health determinants across scales, starting with individual- and family-level factors (e.g., what we eat, the products we use, whether to breastfeed or rely on formula), and extending outward to consider community factors (e.g., proximity to pollution sources, access to fresh food and safe outdoor areas, local air and water quality in both urban and rural settings), and, finally, to national and global factors (e.g., climate change and its diverse ramifications for children’s health).

We have long appreciated that the most vulnerable children are often those living in the communities most in need of environmental intervention, but we have lacked effective, coordinated means to fix the problem. Now, with increased attention on community factors that contribute to children’s health and well-being, we are realizing that effective environmental and public health intervention requires the collaborative efforts of decision makers across all sectors of society. Therefore, we need research to inform governmental sectors that act to prevent exposures and risks and to preserve natural environments, commercial sectors that design and repair the built environment and provide safe products, and public health sectors that create policy to prevent and treat childhood diseases. Recent articles focusing on multiple consequences of fracking, the complexity of indoor air pollution, the benefits of walkability in neighborhoods and access to green and blue spaces with respect to reducing obesity and improving neurobehavioral function in children, and how climate change may affect asthma risks illustrate the importance of decisions about the built and natural environments beyond those designed to reduce chemical exposures.

This year’s Collection includes numerous reports based on cohort studies and surveys from around the globe. These involve pregnant women and children in Canada, Mexico, Costa Rica, Brazil, Korea, China, Taiwan, Bangladesh, Tanzania, Belgium, Denmark, Norway, France, Spain, Switzerland, England, Greece, and Yugoslavia, as well as in the United States. Some of these studies, including those under way in the Children’s Environmental and Disease Prevention Research Program funded by the National Institute of Environmental Health Sciences and the U.S. Environmental Protection Agency (U.S. EPA 2015), are longitudinal in nature: They follow children from before birth through early childhood and into school age and adolescence. These and other longitudinal studies are finding associations between early-life exposures and a wide variety of adverse health outcomes, from birth defects and low birth weight to asthma, childhood cancer, neurodevelopmental problems (e.g., autism, attention deficit/hyperactivity disorder, impaired cognitive function), and metabolic problems associated with obesity (e.g., hypertension, diabetes).

Comparing the latest *EHP* Children’s Health Collection with past Collections, we see a noteworthy transition from an emphasis on a single chemical as it relates to a single disease outcome, to a broader analysis of complex exposures (e.g., measuring multiple chemicals, evaluating both chemical and social stressors) in association with multiple disease outcomes. For example, recent research related to neurodevelopment continues existing lines of research on the adverse effects of metals while expanding to consider effects of cigarette smoke, air pollution, pesticides, phthalates, perfluorinated compounds, and/or organochlorines. Furthermore, given the growing problem of childhood obesity, recent papers have sought associations between a wide range of environmental contaminants and measures of body weight, growth, obesity, hypertension, and diabetes. We also see emerging interest in how placental function may modulate fetal exposures, and how social and behavioral factors (including diet), as well as the child’s microbiome, may influence childhood exposures and responses. As children in cohort studies age, researchers will be able to analyze how cumulative exposures to multiple chemicals and other stressors, both during critical windows of development and collectively, contribute to children’s health as it changes across the life course.

Studies in the 2015 Collection, as in years past, evaluated biomarkers in pregnant women and children, not only in the context of identifying avenues for early-life exposures, but also for characterizing molecular initiating events operating on one or more critical developmental processes in the pathway toward disease. For example, several studies reported changes in DNA methylation of specific genes in association with exposures to metals and cigarette smoke during early development. In addition to increased attention on epigenetic mechanisms of disease causation, we see continued focus on endocrine-mediated developmental effects, and oxidative stress as a common pathway to childhood disease.

Biomonitoring data from national surveys and cohort studies, combined with advances in analytical chemistry, continue to define the maternal and child exposome (the totality of environmental exposures over the life course). The plethora of biomonitoring data, in turn, requires more sophisticated approaches for interrogation and analysis. In response, the children’s environmental health community is calling for international cooperation to build human exposure databases and combine biomarker studies. The ESCAPE (European Study of Cohorts for Air Pollution Effects) (ESCAPE 2014) and NewGeneris (CREAL 2011) programs are combining birth cohorts from across Europe and working toward harmonized data collection and sample processing. This approach not only increases statistical power to detect associations, but also shows how exposures vary from country to country. Related efforts in the United States are under way in the Children’s Health Exposure Analysis Resource (CHEAR) program (NIEHS 2015), which is designed to provide tools for comprehensive children’s exposure assessment and data analysis, and through a new National Institutes of Health initiative called Environmental Influences on Child Health Outcomes (ECHO) (Schmidt 2015), which is designed to support longitudinal birth cohorts, build data and tissue repositories, and develop better analytical tools.

Thus, the year ahead holds great promise for new research, and we invite you to submit your best manuscripts to *EHP*. Looking ahead to our preparations for the Children’s Health Collection 2016, we also welcome your suggestions for making its presentation ever more informative and useful.
